# Spared Nerve Injury Causes Sexually Dimorphic Mechanical Allodynia and Differential Gene Expression in Spinal Cords and Dorsal Root Ganglia in Rats

**DOI:** 10.1007/s12035-021-02447-1

**Published:** 2021-07-30

**Authors:** F.H.G. Ahlström, K. Mätlik, H. Viisanen, K.J. Blomqvist, X. Liu, T.O. Lilius, Y. Sidorova, E.A. Kalso, P.V. Rauhala

**Affiliations:** 1 Department of Pharmacology, Faculty of Medicine, University of Helsinki, Haartmaninkatu 8 (Biomedicum 1), 00014 Helsinki, Finland; 2 Individualized Drug Therapy Research Program, Faculty of Medicine, University of Helsinki, Haartmaninkatu 8 (Biomedicum 1), 00014 Helsinki, Finland; 3 Laboratory of Molecular Neuroscience, Institute of Biotechnology, HiLIFE, University of Helsinki, Viikinkaari 5D, 00014 Helsinki, Finland; 4 Systems Biology/Pathology Research Group and Proteomics Unit, Institute of Biotechnology, HiLIFE, University of Helsinki, 00014 Helsinki, Finland; 5 Department of Anaesthesiology, Intensive Care Medicine and Pain Medicine, Helsinki University Hospital and University of Helsinki, Stenbäckinkatu 9, P.O. Box 440, 00029 Helsinki, Finland; 6 Center for Translational Neuromedicine, Faculty of Health and Medical Sciences, University of Copenhagen, Norre Allé 14, DK-2200 Copenhagen, Denmark; 7 Department of Clinical Pharmacology, University of Helsinki and Helsinki University Hospital, Tukholmankatu 8 C, 00014 Helsinki, Finland; 8 SleepWell Research Programme, Faculty of Medicine, University of Helsinki, Haartmaninkatu 3, 00014 Helsinki, Finland; 9 Emergency Medicine, University of Helsinki and Department of Emergency Medicine and Services, Helsinki University Hospital, Haartmaninkatu 4, 00290 Helsinki, Finland

**Keywords:** Neuropathic pain, Rat, Sex-differences, Behaviour, Transcriptomics, Proteomics

## Abstract

Neuropathic pain is more prevalent in women. However, females are under-represented in animal experiments, and the mechanisms of sex differences remain inadequately understood. We used the spared nerve injury (SNI) model in rats to characterize sex differences in pain behaviour, unbiased RNA-Seq and proteomics to study the mechanisms. Male and female rats were subjected to SNI- and sham-surgery. Mechanical and cold allodynia were assessed. Ipsilateral lumbar dorsal root ganglia (DRG) and spinal cord (SC) segments were collected for RNA-seq analysis with DESeq2 on Day 7. Cerebrospinal fluid (CSF) samples for proteomic analysis and DRGs and SCs for analysis of IB-4 and CGRP, and IBA1 and GFAP, respectively, were collected on Day 21. Females developed stronger mechanical allodynia. There were no differences between the sexes in CGRP and IB-4 in the DRG or glial cell markers in the SC. No CSF protein showed change following SNI. DRG and SC showed abundant changes in gene expression. Sexually dimorphic responses were found in genes related to T-cells (*cd28*, *ctla4*, *cd274*, *cd4*, *prf1*), other immunological responses (*dpp4*, *c5a*, *cxcr2* and *il1b*), neuronal transmission (*hrh3*, *thbs4*, *chrna4* and *pdyn*), plasticity (*atf3, c1qc* and *reg3b*), and others (*bhlhe22*, *mcpt1l, trpv6*). We observed significantly stronger mechanical allodynia in females and numerous sexually dimorphic changes in gene expression following SNI in rats. Several genes have previously been linked to NP, while some are novel. Our results suggest gene targets for further studies in the development of new, possibly sex-specific, therapies for NP.

**Supplementary Information:**

The online version contains supplementary material available at 10.1007/s12035-021-02447-1.

## Introduction

Neuropathic pain (NP) affects 7–8% of the adult population [1]. In most cases, it is refractory to the majority of clinically used drugs [2]. Gender differences in NP or responses to drugs used in NP have not been systematically assessed. Female subjects have often been excluded from clinical trials [3], even though NP, as well as chronic pain in general, is more prevalent among women [4, 5]. However, a systematic review concerning studies of human experimental pain found no consistent pattern in gender differences [6]. Several psychological and sociocultural phenomena may influence pain perception differently in the two genders. Therefore, non-human studies are needed to uncover the underlying pathophysiologic mechanisms explaining possible sex differences in pain perception [7].

Preclinical studies indicate that the mechanisms in the pathophysiology of NP may differ between males and females. Female rats develop NP relatively more often than males after partial sciatic nerve ligation (SNL) [8]. On the other hand, studies comparing females and males with chronic constriction injury (CCI) in rats and spared nerve injury (SNI) in mice do not report sex differences in baseline behaviour [9, 10], though there is evidence of time-dependent sex differences in mouse behaviour with CCI [11].

As microglial activation has been demonstrated in both sexes following peripheral nerve injury (PNI), it has been suggested that the pathways leading to NP could diverge at the microglial level [9, 12–15]. In support of this hypothesis, inhibition of pro-inflammatory microglial activation attenuates mechanical allodynia following PNI in male, but not female, mice and rats [9, 10]. In females, on the other hand, allodynia seems to be inducible without microglial activation, perhaps relying preferentially, but not exclusively, on mechanisms involving adaptive immune cells instead [10]. T lymphocytes have been suggested to be important in the induction of allodynia in female rats. These cells can also infiltrate the spinal cord (SC) following PNI and play a role in pain hypersensitivity [10, 16]. These and other neuroimmune processes, also studied in humans, are of great interest in explaining persistence of NP [17].

Recent advances in analysing unbiased transcriptomics data have been used to elucidate the mechanisms of NP. Zhou et al, have analysed the transcriptome of spinal cords following SNI in male rats, at several time points for up to day 14, showing several changes in mRNA and lncRNA expression [18]. The authors also called for future sex-specific analyses. Furthermore, RNA-Seq of rat dorsal root ganglia (DRG) in both sexes has revealed both common and sex-specific differences in gene expression following CCI, particularly in synthesis of cytokines, growth factors and neurotransmitters. Several sex-specific changes have also been found in the MAPK pathway [19] which has been linked to NP [20, 21].

As the sexual dimorphism of pain and the sex-specific role of glial and immune cells in NP are still inadequately understood, we set out to quantify sex differences in the behaviour of rats following SNI and to discover novel genes and proteins, as well as biological processes, displaying sexually dimorphic responses in NP. In addition to immunohistochemistry (IHC), we used unbiased RNA-Seq and proteomics, in order to provide a novel insight into the molecular makings of sex differences in NP, and possible targets for future therapeutic interventions for NP.

## Materials and Methods

This study consisted of two separate experiments, I and II. Experiment I continued for 21 days, while Experiment II, concentrating on an earlier phase of development of NP, lasted for 7 days. We conducted behavioural tests in both experiments. In Experiment I, we collected cerebrospinal fluid (CSF) and samples for IHC (as detailed below). In Experiment II, we analysed DRG and SC, utilising RNA-Seq.

### Animals

In Experiment I, we used 20 male and female Sprague-Dawley rats, and 24 males and females in Experiment II. The animals were housed at the Laboratory Animal Centre of Biomedicum Helsinki in individually ventilated cages, with two animals per cage. Standard food and water were provided ad libitum. The facility had 12-h long alternating light and dark conditions. Animals rested for 2 weeks before baseline tests. The animals were habituated to the behavioural testing protocol for three consecutive days prior to the baseline tests. Great care was taken to minimize animal stress. All rats were handled in a similar way. The animals were nine weeks of age at the time of the baseline tests; mean female weight was 218 g (95% CI [213, 224]) and mean male weight was 341 g (95%, CI [332, 348]). The 3Rs principle was applied and ARRIVE guidelines were followed during the study [22, 23]. All procedures were approved by the Regional State Administrative Agency for Southern Finland (ESAVI-9697/04.10.07-2017).

The law on the Protection of Animals Used for Scientific or Educational Purposes (497/2013) and Directive 2010/63/EU on the protection of animals used for scientific purposes Text with EEA relevance were followed fully.

### Spared Nerve Injury Surgery

The SNI model of NP produces consistent, prolonged, and substantial changes in thermal and mechanical sensitivity on the lateral aspect of the hind paw, mimicking several features of clinical NP [24].

SNI surgery was performed on half of the animals of each sex, with the other half undergoing sham surgery. Anaesthesia was induced and maintained with 4% and 2% isoflurane, respectively.

In the animals undergoing SNI surgery, the right sciatic nerve, originating from spinal segments L4-6 [25], was exposed by cutting the skin and subcutaneous tissue using a scalpel. The muscles overlying the sciatic nerve were prepared and cut through by spreading with scissors in the direction of the muscle fibres. The tibial and fibular divisions of the sciatic nerve were ligated once with fine 8-0 non-absorbable suturing silk, after which the divisions were cut distally. Utmost care was taken to leave the sural branch of the sciatic nerve intact. The muscle cut was sutured with absorbable 4-0 polyglactin suturing thread. The skin was sutured using non-absorbable 4-0 thread.

In the sham surgery animals, the right sciatic nerve was identically exposed. The nerve was left intact, and the surgical wound was sutured as in the animals undergoing SNI surgery.

### Behavioural Tests

Behavioural testing was conducted before surgery, seven days postoperatively in both studies, and 21 days after surgery in Experiment I. We used von Frey filaments [26] and acetone testing [27] to assess tactile nociception and cold allodynia, respectively. Investigators were blinded to treatment groups during behavioural tests, which were always conducted in the aforementioned order. Animals were tested in translucent plastic boxes with a floor of metal grids through which filaments and acetone were introduced. Animals were habituated to test conditions prior to tests with von Frey filaments.

### Von Frey Assay

We used von Frey filaments to assess static mechanical allodynia, conducted through C-fibres [28]. We used filaments calibrated to apply forces of 0.4, 1, 2, 4, and 6 g to prod each animal five times per filament on the lateral plantar aspect of both hind paws. Prods were conducted one second apart on individual animals, with six animals being prodded consecutively, before repeating with the next filament. A forceful retraction of the hind paw was recorded as a positive response. In addition to reporting responses for individual filaments, the total number of positive responses from the filaments was summed.

### Acetone Testing

Assessment of cold allodynia was conducted by spraying acetone on both hind paws. Cold sensitivity is mediated by both C- and Aδ-fibres [29]. Each hind paw was sprayed and tested five times, with a minute-long period of observation and another minute-long pause between sprays. A positive response was recorded if the animal clearly attended to its paw during observation, excluding responses during the first five seconds.

### Sample Extraction

#### Experiment I

We extracted the tissue samples after completion of the last behavioural tests. We perfused the animals transcardially under isoflurane anaesthesia, first for 3 min with phosphate-buffered saline (PBS), and then for 15 min with 4% paraformaldehyde (PFA) solution. We collected CSF samples (ca. 100 μl/sample) with a cervical puncture under 2% isoflurane anaesthesia.

We excised a segment of SC, corresponding to the L4-L5 level, and bilateral L4-L5 DRG and immediately submerged them in 4% PFA-solution. We changed the PFA solution after one and three days, after which the samples were embedded in blocks of paraffin wax.

#### Experiment II

We perfused the animals transcardially under isoflurane anaesthesia with PBS after the last behavioural tests. We excised the ipsilateral SC segments L4-L5 and the corresponding ipsilateral DRG, snap-froze them in liquid nitrogen and stored them at −70 °C.

#### Immunohistochemistry

For the IHC analyses, we prepared sections of 10 μm thickness from L4 and L5 segments of lumbar regions of SC and of 7 μm from DRG, after the initial extraction.

We probed the SC sections with antibodies for IBA1 (1:1000, Cat# 019-19741, Wako, Richmond, VA, USA) or GFAP (1:400, Cat# G-3893, Sigma-Aldrich, St. Louis, MO, USA). DRG sections were probed with antibodies for CGRP (1:10,000, Cat# T-4032, Peninsula Laboratories, San Carlos, CA, USA) or FITC-conjugated IB-4 (1:200; Cat# FL-1201, Vector Labs, CA, USA). Bound antibodies were visualized using anti-rabbit and anti-mouse biotinylated secondary antibodies and the VECTASTAIN ABC HRP Kit (Cat PK-6101, PK-4002 Vector Laboratories, Burlingame, CA, USA) with 3,3’-diaminobenzidine (DAB) as a chromogene, according to the manufacturer’s instructions. DAB-stained slides were imaged using the 3DHISTECH Scanner (3DHISTECH Ltd, Budapest, Hungary) at the scanning service provided by Biocenter Helsinki (http://www.biocenter.helsinki.fi/bi/histoscanner/index.htm l). Fluorescent-labelled slides were imaged using a Leica DM6000B microscope (Leica, Germany). Images of SC sections were analysed with Matlab R2014b software (Mathworks, Natick, MA, USA). The number of IBA1- or GFAP-positive cells was quantified using in house-developed scripts with manually set size and intensity thresholds, as described previously [30]. The number of cells was normalized to the area of analysed tissue. Since the morphology of glial cells is changed upon activation, we also analysed the area covered by immunopositive stainings, determined as the total number of pixels with intensity above the selected threshold and normalized to the area of analysed tissue in pixels. All these parameters were calculated for the left and right dorsal and ventral horns (DH and VH). The resulting data are presented as percentages of the respective data from the intact side to minimize inter-slide variation. For each animal and region studied, three (in most cases) non-consecutive sections were quantified, and the results averaged for statistical analysis. The following exclusions were made from the IHC data: the data for one sham-operated female were excluded from statistical analysis of L4 IBA1 group due to severe damage of the tissue in the analysed region; in the analysis of GFAP expression, outliers were excluded using the ROUT method with Q = 1% (1 animal per group). DRG sections were mounted in Imu-mount or Coverquick 2000 (Q PATH, Cat#05547530) mounting media. Slides were imaged with a fluorescence microscope (Zeiss Imager M2 Axio, Carl Zeiss, Germany) or scanned using 3DHISTECH. For each DRG, the number of the cells positive for the specific marker was counted from three random sections and normalized to the total number of neurons in the corresponding section. The data for each DRG were averaged and used for statistical analysis.

### Proteomics

Cysteine bonds were reduced with 0.05M TCEP-HCl (Tris(2-carboxyethyl) phosphine hydrochloride salt, #C4706 Sigma-Aldrich, USA) for 20 min at 37 °C and alkylated with 0.15M iodoacetamide (#57670 Fluka, Sigma-Aldrich, USA) at room temperature. Samples were digested by adding 1 μg trypsin (Sequencing Grade Modified Trypsin, V5111, Promega) and leaving overnight at 37 °C. After digestion, peptides were quenched with 10% trifluoroacetic acid (TFA) and purified with C18 microspin columns (Harvard Apparatus, USA) eluting the samples with 0.1% TFA in 50% acetonitrile (ACN). The dried peptides were reconstituted in 30 μl 0.1% TFA in 1% ACN (buffer A).

Liquid chromatography-tandem mass spectrometry (LC-MS/MS) analysis was carried out on an EASY-nLC 1000 (Thermo Fisher Scientific, Germany) connected to a Q Exactive hybrid mass spectrometer (Thermo Fisher Scientific, Germany) with nano-electrospray ion source (Thermo Fisher Scientific, Germany). The LC-MS/MS samples were separated using a two-column setup consisting of a 2 cm C18 Pepmap column (#164946 Thermo Fisher Scientific, Germany), followed by 15 cm C18 Pepmap analytical column (#164940 Thermo Fisher Scientific, Germany). The linear separation gradient consisted of 5% buffer B in 5 min, 35% buffer B in 60 min, 80% buffer B in 5 min and 100% buffer B in 10 min, at a flow rate of 0.3 μl/min (buffer A: 0.1% TFA in 1% acetonitrile; buffer B: 0.1% TFA acid in 98% acetonitrile). 4 μl of sample was injected per LC-MS/MS run and analysed. Each animal sample, *n* = 8 in males per group and *n* = 6–7 in females per group, was run and analysed separately. Full MS scan was acquired with a resolution of 70 000 at normal mass range in the Orbitrap analyser. The method was set to fragment the 10 most intense precursor ions with HCD. LC-MS/MS analysis was performed using the Xcalibur and LTQ Tune Software.

Acquired MS2 scans were searched against the *Rattus norvegicus* protein database using the Sequest search algorithms in Thermo Proteome Discoverer. The allowed mass error for the precursor ions was 15 ppm and, for the fragment, 0.05 Da. A static residue modification parameter was set for carbamidomethyl +57,021 Da (C) of cysteine residue. Methionine oxidation was set as dynamic modification +15,995 Da (M). Only full-tryptic peptides with a maximum of 1 missed cleavage were considered.

### RNA Extraction and Sequencing

Extraction and sequencing of RNA from SC and DRG tissue were conducted at the Functional Genomics Unit, Biomedicum Helsinki. Precellys 24 was used for lysis and homogenization. Illumina’s ScriptSeq Complete Gold Kit (Human/Mouse/Rat) was used for library preparation. Sample quality was assessed with Agilent 2100 Bioanalyzer. Ribosomal RNA was removed using Ribo-ZeroTM Gold rRNA Removal Kit. Sequencing was carried out using Illumina’s NextSeq High Output 1 x 75 bp kit. All samples, *n* = 7 in all groups, were sequenced and analysed separately.

### Statistical Analyses

#### Behavioural Tests

The behavioural data were analysed using GraphPad’s Prism 7 (La Jolla, CA, USA). Behavioural data of the groups at the different time points were analysed using two-way ANOVA with Holm-Sidak correction for multiple comparisons. An adjusted p value of < 0.05 was considered statistically significant throughout the study in all analyses, with * = *p* < 0.05, ** = *p* < 0.01, *** = *p* < 0.001 and **** = *p* < 0.0001. Standard errors of the mean were also reported.

#### Immunohistochemistry

All quantitative data were analysed using multiple *t*-tests to compare parameters of interest between males and females in Graphpad Prism 6 (La Jolla, CA, USA). Correction for multiple t-tests was done using the Holm-Sidak method (*α* = 0.05). Each row was analysed individually without assuming a consistent standard deviation (SD). All data are presented as mean ± SEM.

### Proteomics

The peptide-spectrum match-values of the proteomics data were used to calculate the relative abundance (LFQ-intensity) of proteins using MaxQuant [31]. LFQ-intensity for all individual sample values were then compared in GraphPad’s Prism 7 using multiple *t*-tests with Holm-Sidak multiple comparisons correction to analyse differential expression of proteins in the treatment groups.

### RNA Differential Expression—Spinal Cords and Dorsal Root Ganglia

RNA differential expression analysis was carried out at the Functional Genomics Unit, Biomedicum Helsinki. Quality of samples was considered adequate, using the FastQC package [32]. Light quality trimming of the data was carried out using the Trimmomatic software [33]. After this, the reads were aligned to the Rnor_6.0 genome from Ensembl.org [34] using STAR [35]. Read count tables were created using the featureCounts software [36], after which differential expression was calculated using the DESeq2 package [37].

A cut-off value for False Discovery Rate (FDR) of < 0.05 was used to select differentially expressed genes. Identification of genes with sexually dimorphic responses to SNI was made by matching genes that showed differential expression (DE) in only one sex due to SNI or genes that showed no sexually dimorphic expression in the sham-surgery groups but did so after SNI. In order to identify known pain genes with sexually dimorphic responses to SNI, pain genes were retrieved from the rat-centric PainNetworks database [38] and compared with sexually dimorphically expressed genes. Additionally, in order to identify novel genes of interest, sexually dimorphically expressed genes were ordered based on their sex fold-change (FC) ratio, which we defined as (*FemaleSNI*/*FemaleSham*)/(*MaleSNI*/*MaleSham*), with the cut-off FC ratio being 2.5 < × < 0.4 and 1.5 < × < 0.667 for DRG and SC tissue, respectively. Finally, we manually selected several genes, based on prior research, in order to replicate prior findings and to find genes of interest for further investigation. Genes with a sexually dimorphic response that showed an inconsistent pattern of expression across samples in each group were manually excluded.

Pathway analyses were performed with Advaita iPathwayGuide [39], using FDR < 0.05 and log_2_ FC > 0.6 as input criteria for the data. We corrected the pathway analysis results using FDR, and Gene Ontology biological processes, using Elim Pruning (EP) with a p value of < 0.05 as a limit for statistical significance.

## Results

### Behavioural Tests—Experiments I and II

#### von Frey assay

**Fig. 1:**
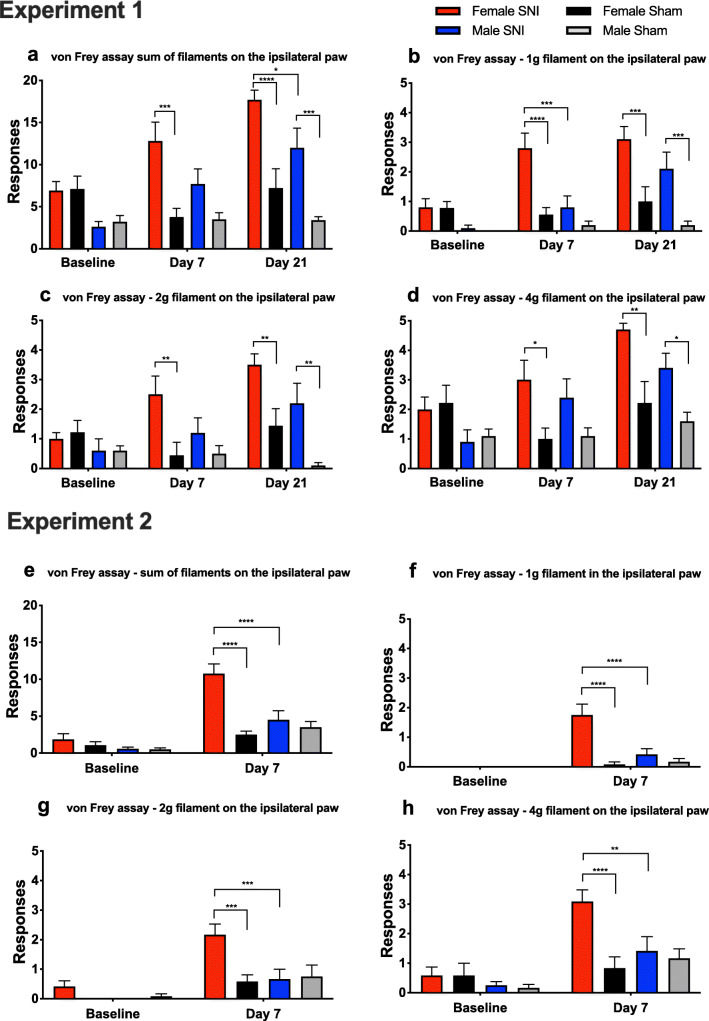
Results from the von Frey assay. Conducted on the ipsilateral paw in Experiment I at baseline prior to SNI surgeries, on Days 7 and 21 and in Experiment II at baseline prior to surgeries and on Day 7. Mean responses and SEM are reported. Five trials with five von Frey filaments (0.4, 1, 2, 4 and 6g) were conducted. Positive responses in the experiments were summed and are shown in Fig. 1a and 1e, respectively. The maximum number of responses was 25. Positive responses for filaments 1g, 2g and 4g in the experiments are reported in Fig. 1b, c and d, and in Fig. 1f, g and h, respectively. *n* = 9 or 10 rats per group (Experiment I) and *n* = 12 rats per group (Experiment II). 2-way-ANOVA with Holm-Sidak correction, * *p* < 0.05, ** *p* < 0.01, *** *p* < 0.001, **** *p* < 0.0001

In Experiment I, responses on the ipsilateral side were similar during the baseline test in all groups when results from the five filaments were summed. On Day 7 after SNI surgery, the responses had significantly increased in the female group (F SNI vs. Sham *p* < 0.001), but not in male rats (Fig. 1a). On Day 21, the number of responses had further increased in the SNI groups, with both SNI groups showing robust increases (M SNI vs. Sham *p* < 0.001; F SNI vs. Sham *p* < 0.0001). Females had more positive responses than males on Day 21 (*p* < 0.05).

When inspecting responses recorded with individual filaments, lower threshold filaments showed larger sex differences on Day 7 (M vs. F SNI 1 g *p* < 0.001, 2 g *p* > 0.05 and 4 g *p* > 0.05) but not on Day 21 (Fig. 1b, c and d).

Experiment II provided largely similar results, replicating the finding that females responded more often to the von Frey filaments following SNI. Stimulation of the ipsilateral side showed no difference at baseline between any groups. On Day 7 after surgery, females showed an increased number of responses compared with baseline (*p* < 0.0001), while males did not (Fig. 1e). A significant sex difference was observed, with females showing more frequent responses, indicating greater mechanical allodynia (*p* < 0.0001). Results on individual filaments showed an even more robust gender difference, as stimulation with lower threshold filaments resulted in more responses in females on Day 7 (M vs. F SNI 1g *p* < 0.0001, 2g *p* < 0.001 and 4g *p* < 0.01) (Fig. 1f, g, and h).

#### Acetone Test

**Fig. 2:**
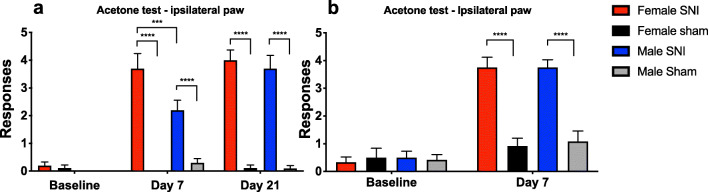
Results from the acetone test conducted on the ipsilateral paw in Experiment I (Fig. 2a) at baseline prior to spared nerve injury (SNI) surgeries, on Days 7 and 21 and II (Fig. 2b) at baseline prior to surgeries, and on Day 7. Mean responses and SEMs are reported. Five trials with acetone were conducted on each animal. Positive responses were summed. *n* = 9 or 10 rats per group (Experiment I) and *n* = 12 rats per group (Experiment II). 2-way-ANOVA with Holm-Sidak correction, * *p* < 0.05, ** *p* < 0.01, *** *p* < 0.001, **** *p* < 0.0001

In Experiment I, both males and females in the SNI groups demonstrated robust increases in responses to the acetone test on the ipsilateral side after both Days 7 and 21, with significant differences for M SNI vs. Sham and F SNI vs. Sham (*p* < 0.0001) (Fig. 2a). The sham-operated animals showed no change in responses during the experiment. Female SNI rats showed more frequent responses than male SNI rats on Day 7 (*p* < 0.001), but this difference had disappeared by Day 21 when male responses had risen to a level similar to that of the females.

In Experiment II, however, while both SNI groups showed more frequent responses than the respective sham groups on Day 7 (M SNI vs. Sham and F SNI vs. Sham *p* < 0.0001), no difference in responses between SNI-treated males and females could be demonstrated at this time point (Fig. 2b). Identical testing on the contralateral side revealed no changes between the SNI- and sham-treated rats in the positive responses to acetone testing in Experiment I on baseline, on Day 7 or in Experiment II. On Day 21 of Experiment I, M Sham vs. F Sham and Female SNI vs. F Sham showed a slight change (*p* = 0.0267) (Supplementary figure 1).

### Immunohistochemistry—Experiment I on Day 21

#### The Expression of IBA1 in the SC of Sham- and SNI-Operated Animals

**Fig. 3:**
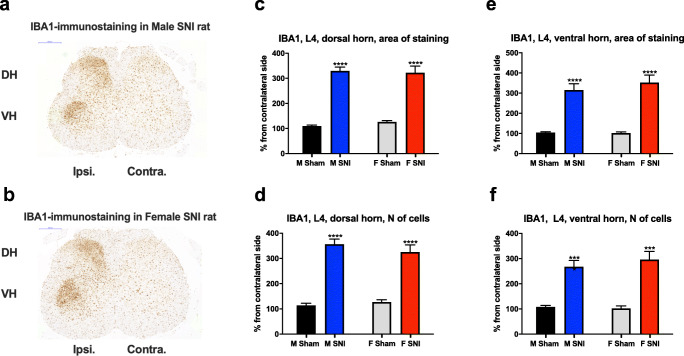
Analysis of IBA1 expression in the L4 region of the spinal cord (SC). Sham- and SNI-operated male (M) and female (F) rats on day 21 day after baseline tests. Figure 3a and b shows representative images of IBA1-immunostained L4 SC section of a male (3a) and a female (3a) rat. The dotted line outlines the analysed areas. 3c shows the area covered by IBA1-positive cells in the dorsal horn (DH); 3d shows the number of IBA1-positive cells in the DH; 3e shows the area covered by IBA1-positive cells in the vental horn (VH); and 3f the number of IBA1-positive cells in VH. All data normalized to the size of the analysed area (in pixels) and expressed as percentage of the contralateral side. *** *p* < 0.001, **** *p* < 0.0001, Student’s t-test with Holm-Sidak correction for multiple comparisons. *n* = 8–10 rats per group. Contra. = contralateral side; Ipsi. = ipsilateral side; *n* of cells = number of cells

SNI rats showed a significantly larger area of IBA1 staining on the ipsilateral side of L4 SC tissue in ventral horns (VHs) and dorsal horns (DHs) in both males (VH 201%, DH 201%) and females (VH 244%, DH 156%) than the respective sham groups (*p* < 0.05). Similarly, the number of IBA1-positive cells increased in both SNI males and females in VHs and DHs, compared with sham rats (*p* < 0.05). We observed no differences between the sexes in the area of IBA1 staining or in the number of IBA-1 positive cells in the L4 region of the SC in either sham- or SNI-operated animals (*p* > 0.05) (Fig. 3).

#### The Expression of GFAP in the SC of Sham- and SNI-Operated Animals

We also compared the number and the area covered by GFAP-positive cells in the L4 region of SC of sham- and SNI-operated animals of both sexes. In general, SNI increased the expression of GFAP in the SC of both females and males, but to a lower extent than that of IBA1 (*p* < 0.05), with the exception of the number of cells in males in the VH (*p* > 0.05). The results are detailed in Table 1. We observed no statistically significant differences between sexes in the expression of GFAP in either the VHs or the DHs of sham-operated animals, or in the DH of the L4 region of the SC of SNI-operated animals.

**Table 1:** GFAP-staining in L4 spinal cord (SC) tissue after SNI compared with sham in male and female rats. All data normalized to the size of the analysed area (in pixels) and converted to percentage of the respective values on the contralateral side. *p* * < 0.05, ** *p* < 0.01, *** *p* < 0.001, **** < 0.0001, *p* > 0.05 = NS, Student’s *t*-test with Holm-Sidak correction for multiple comparisons. *n* = 8–10 rats per group. *DH* dorsal horn; *FC* fold-change; *L* lumbar; *NS* not significant; *VH* ventral horn

**GFAP**		**Female SNI vs. Sham**		**Male SNI vs. Sham**	
**Measure**	**Tissue**	**FC**	**Statistics**	**FC**	**Statistics**
area of staining	L4 DH	1.3	t(17) = 3.6, p < 0.01	1.3	t(18) = 2.9, p < 0.01
number of cells	L4 DH	1.3	t(17) = 2.4, p < 0.05	1.4	t(18) = 3.1, p < 0.01
area of staining	L4 VH	1.9	t(17) = 4.8, p < 0.001	1.3	t(17) = 2.3, p < 0.05
number of cells	L4 VH	1.83	t(17) = 4.3, p < 0.001	NS	(p > 0.05)

#### The Expression of CGRP and IB4 in the DRG of Sham- and SNI-Operated Animals

**Fig. 4:**
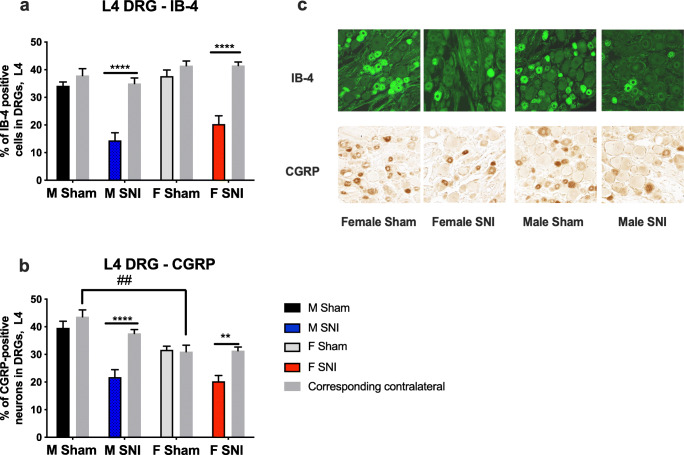
L4 dorsal root ganglion (DRG) immunostaining for IB-4 (a) and CGRP (b) after SNI in male (M) and female (F) rats and representative images of staining in L4 (c). **** *p* < 0.001, ** *p* < 0.01 by Student’s *t*-test with Holm-Sidak correction for multiple comparisons and ANOVA. ## *p* < 0.01 by ANOVA. *n* = 10

IB4-immunostaining revealed a 59% fewer IB4-positive cells in male and 51% fewer in female SNI-operated rats on the ipsilateral side than on the contralateral side (*p* < 0.05) (Fig. 4). No sex differences between the ipsilateral sides of the sham-operated, or between the contralateral sides of SNI- or sham-operated rats, were observed.

Immunostaining for CGRP (Calcitonin gene-related peptide) in L4 DRG in males demonstrated 42% fewer (*p* < 0.05) CGRP-positive cells on the ipsilateral side than on the contralateral side, following SNI (Fig. 4). Similarly, females showed 35% fewer CGRP-positive cells on the ipsilateral side. Neither male nor female shams showed a difference between the ipsi- and contralateral sides. A sex difference in immunostaining for CGRP was found when comparing contralateral male shams with female shams (*p* < 0.05), a difference not observed between, for example, male contralateral SNI rats and female contralateral shams.

#### Proteomics of CSF—Experiment I on Day 21

No protein in the CSF demonstrated a change in amount after SNI, compared with sham surgery, in either sex. Six proteins were sexually dimorphically expressed in either sham or SNI animals (A1BG, VTN, AFM, LOC259246;RGD1566134;LOC298116;LOC688457;LOC298111, KNG1L1 AND KNG1;MAP1: *p* < 0.05) (Table 2 and supplementary table 1). A1BG was not expressed at all in males and the protein group LOC259246;RGD1566134;LOC298116;LOC688457;LOC298111 was not expressed in females.

**Table 2:** Relative amounts (LFQ intensity) of proteins with differences in one or more comparisons in rat cerebrospinal fluid (CSF) 21 days after SNI. Male (M) groups: *n* = 8; Female (F) groups: *n* = 6–7. Multiple *t*-tests with Holm-Sidak correction * = *p* < 0.05 ** = *p* < 0.01, *** = *p* < 0.001 **** *p* < 0.0001, *p* > 0.05 = NS. *A1BG* Alpha-1B-glycoprotein; *VTN* vitronectin; *AFM* afamin; LOC259246;RGD1566134;LOC298116;LOC688457;LOC298111 Alpha-2u globulin-associated proteins; *KNG1L1* Kininogen 1-like 1; KNG1;MAP1 Kininogen 1;T-kininogen 1. *NFP* no female protein; *NMP* no male protein; *NS* not significant

CSF proteins with differential amounts				
Protein name	$\frac{M\ SNI}{M\ Sham}$	$\frac{F\ SNI}{F\ Sham}$	$\frac{F\ SNI}{M\ \boldsymbol{SNI}}$	$\frac{F\ Sham}{M\ Sham}$
A1BG	NMP_NS_	0,80_NS_	NMP ****	NMP**
AFM	1,00_NS_	0,85_NS_	0,48 *	0,57_NS_
KNG1L1	1,24_NS_	1,41_NS_	2,79_NS_	2,45 **
KNG1; MAP1	1,38_NS_	1,18_NS_	2,11_NS_	2,46 **
LOC259246; RGD1566134; LOC298116; LOC688457; LOC298111	0,8_NS_	NFP_NS_	NFP *	NFP_NS_
VTN	0,87	1,29	0,83_NS_	0,57 *

#### Spinal Cord Transcriptomics—Experiment II on Day 7

**Fig. 5:**
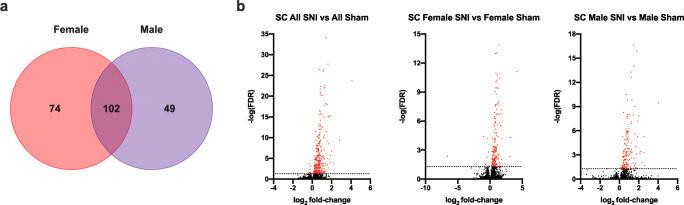
Venn diagram (a) and volcano plots (b) of RNA-Seq data in rat spinal cord (SC) following spared nerve injury (SNI). All genes included in the Venn diagram have a FDR < 0.05. Volcano plots report log_2_ fold-change and negative logarithmic (base 10) False Discovery Rate (FDR). Dotted line at -log( FDR 0.05), red symbols -log(FDR<0.05). Ipsilateral L4-L5 SC segment on day 7 after SNI and Sham-surgeries. All SNI vs. All Sham: *n* = 14 (50% male and female), Male SNI vs. Male Sham and Female SNI vs. Female Sham: *n* = 7. DESeq2 utilized for DE analysis

In Experiment II, analyses of the SC transcriptome showed 328 transcripts with altered expression (FDR < 0.05), when comparing all SNI animals with all sham animals. The greatest upregulation occurred in *Cxcl13*, *Loc299282* and downregulation in *Hoxd9* and *Rpe65*. These and the other largest changes in this comparison are reported in Table 3. When comparing the changes after SNI in females and males separately, 176 and 151 genes were differentially expressed, respectively (FDR < 0.05), 102 genes were altered in both sexes (Fig. 5). In females, the largest single gene upregulation and downregulation occurred in *Cxcl13* and *Amot*, respectively. The largest upregulation in males occurred in *Cxcl13*, no significant downregulation was observed in the male SC (FDR < 0.05) (Table 3 and supplementary table 2a).

**Table 3:** Log_2_ fold-change (FC) of 25 genes with the largest and smallest DE (differential expression) per log_2_ FC in rat ipsilateral spinal cords (SC) L4-L5 7 days after SNI with false discovery rate (FDRs). All SNI vs. All Sham: *n* = 14 (50% male and female), Male SNI vs. Male Sham and Female SNI vs. Female Sham: *n* = 7. DESeq2 was utilized for the DE analysis. All reported genes had FDR < 0.05

Protein coding genes with largest upregulation in spinal cords								
All SNI vs. All Sham SC			Male SNI vs. Male Sham SC			Female SNI vs. Female Sham SC		
Gene name	log_2_ FC	FDR	Gene name	log_2_ FC	FDR	Gene name	log_2_ FC	FDR
*Cxcl13*	4.10	2.1E-24	*Cxcl13*	4.02	3.74E-10	*Cxcl13*	4.19	7.77E-12
*Loc299282*	2.82	4.16E-10	*Ankrd1*	2.61	1.92E-02	*Loc299282*	3.15	4.87E-05
*Ankrd1*	2.22	1.36E-04	*Reg3b*	2.55	5.37E-06	*Prg4*	3.08	4.64E-03
*Reg3b*	1.98	1.84E-07	*Loc299282*	2.53	5.34E-04	*Slc26a7*	2.35	2.58E-02
*Atf3*	1.87	2.01E-06	*F10*	2.34	9.39E-03	*Ccl7*	1.88	2.68E-02
*Fcrl2*	1.86	4.28E-16	*Ccl2*	2.21	3.70E-04	*Clec7a*	1.85	6.59E-05
*Tfec*	1.83	8.09E-05	*Atf3*	2.13	6.14E-04	*Mir146a*	1.76	9.12E-03
*Ccl7*	1.80	2.34E-05	*Fcrl2*	2.00	1.34E-08	*Csf2rb*	1.73	7.11E-08
*Loc100910270*	1.74	1.08E-15	*Aoah*	1.88	4.91E-06	*Fcrl2*	1.71	3.27E-06
*Clec7a*	1.68	3.09E-09	*Loc100910270*	1.85	6.49E-08	*Mefv*	1.70	1.77E-07
*Csf2rb*	1.66	6.84E-16	*C1qc*	1.79	1.27E-16	*Loc100910270*	1.62	2.95E-06
*F10*	1.65	1.99E-03	*Il21r*	1.78	7.64E-04	*Il18rap*	1.53	3.68E-02
*C1qc*	1.63	1.83E-28	*Ccl7*	1.73	3.44E-02	*Lilrb3a*	1.49	2.35E-02
*Il18rap*	1.59	9.76E-06	*Mrgprx3*	1.70	3.84E-02	*C1qc*	1.46	4.02E-11
*Aoah*	1.58	5.86E-10	*Cd22*	1.68	1.01E-04	*Ly86*	1.46	1.5E-06
*Ccl2*	1.57	3.27E-04	*Il18rap*	1.65	1.25E-02	*Clec12a*	1.43	2.02E-07
*Clec12a*	1.53	1.05E-18	*Clec12a*	1.62	1.01E-09	*Mx1*	1.41	1.76E-02
*Mefv*	1.46	3.27E-12	*Csf2rb*	1.59	2.16E-06	*Fcgr3a*	1.38	2.33E-08
*C3*	1.42	8.01E-35	*Pbk*	1.56	2.20E-02	*C3*	1.37	1.4E-14
*Cd6*	1.41	4.61E-04	*Clec7a*	1.53	2.72E-03	*Apobec1*	1.35	8.23E-03
*Fcgr3a*	1.40	5.9E-19	*C1qb*	1.51	9.27E-15	*Itgal*	1.32	1.16E-08
*Il21r*	1.32	1.97E-04	*C3*	1.48	2.35E-17	*Aoah*	1.31	2.65E-03
*Loc691141*	1.32	5.86E-03	*Rbm47*	1.47	9.77E-03	*C1qa*	1.26	3.92E-10
*C1qb*	1.32	2.91E-23	*Gpr65*	1.46	4.26E-02	*Cd40*	1.26	4.98E-02
*Mmp3*	1.30	1.39E-02	*Fcgr3a*	1.42	1.11E-08	*Loc103689965*	1.26	3.95E-05
Protein coding genes with largest downregulation in spinal cords								
All SNI vs. All Sham SC			Male SNI vs. Male Sham SC			Female SNI vs. Female Sham SC		
Gene name	log_2_ FC	FDR	Gene name	log_2_ FC	FDR	Gene name	log_2_ FC	FDR
*Hoxd9*	-0.57	4.84E-02	None	-	-	*Ddx3*	-2.09	2.87E-02
*Rpe65*	-0.32	1.97E-04				*Zbtb16*	-1.01	3.95E-05
*Med16*	-0.31	2.74E-02				*Frmpd1*	-0.72	3.18E-02
*Apln*	-0.29	2.21E-02				*Ccdc92*	-0.65	2.79E-02
*Lmln*	-0.25	3.02E-02				*Pcdh20*	-0.46	5.48E-03
*Cacnb2*	-0.19	2.88E-02				*Rpe65*	-0.46	6.59E-05
*Camk2g*	-0.18	4.84E-03				*Cacnb2*	-0.26	4.05E-02
*Amot*	-0.17	6.46E-04				*Amot*	-0.23	1.59E-03
*Gtf3c1*	-0.14	4.03E-02

A total of 10 genes in the rat-centric PainNetworks database were identified as sexually dimorphically expressed following SNI. One of these was prodynorphin (FDR < 0.001) (Table 4). Several other genes also showed sexually dimorphic responses to SNI. Some of these additional genes were chosen for highlighting. Also of interest are *Tyrobp* and *Aif1* (coding for IBA1), both upregulated only in males (FDR < 0.05), (Table 5, and supplementary table 2a). The genes with the largest sex-linked fold-change ratio are shown in Table 6 and supplementary table 2a.

**Table 4:** Mean fold change of normalized counts of genes from the PainNetworks rat-centric database in spinal cord (SC) with sexually dimorphic expression. Ipsilateral L4-L5 SC 7 days after spared nerve injury (SNI) differential expression (DE) analysed with DESeq2. All groups: *n* = 7. False discovery rate < 0.05 = *, < 0.01 = **, < 0.001 = ***, < 0.0001 = **** and > 0.05 = NS

Gene name	Description (Ensembl.org)	$\frac{FSNI/ FSham}{MSNI/ MSham}$	$\frac{MSNI}{MSham}$	$\frac{FSham}{MSham}$	$\frac{FSNI}{FSham}$
*Abcc3*	Canalicular multispecific organic anion transporter 2	0,76	1,53**	1,08	1,17_NS_
*Ccr5*	C-C chemokine receptor type 5	1,08	1,33_NS_	0,98	1,44**
*Ifngr1*	Interferon gamma receptor 1	0,82	1,40**	1,72	1,15_NS_
*Kcnj5*	G protein-activated inward rectifier potassium channel 4	1,30	1,43_NS_	0,79	1,87**
*P2ry12*	Purinergic receptor P2Y12	1,20	1,38_NS_	0,96	1,65****
*Pdyn*	Prodynorphin	0,70	1,93***	1,08	1,34_NS_
*Prkcd*	Protein kinase C delta	1,06	1,19_NS_	0,90	1,27*
*Tlr3*	Toll like receptor 3	1,12	1,14_NS_	0,82	1,27***
*Tlr4*	Toll like receptor 4	0,91	1,40**	1,18	1,28_NS_
*Trpm2*	Transient receptor potential cation channel subfamily M member 2	0,87	1,42***	1,19	1,24_NS_

**Table 5:** Mean fold change of normalized counts of selected genes of interest in spinal cord (SC) with sexually dimorphic expression. Ipsilateral L4-L5 SC 7 days after spared nerve injury (SNI). Differential expression (DE) analysed with DESeq2. All groups: n = 7. False discovery rate < 0.05 = *, < 0.01 = **, < 0.001 = ***, < 0.0001 = **** and > 0.05 = NS

Gene name	Description (Ensembl.org)	$\frac{F\ SNI/F\ Sham}{M\ SNI/M\ Sham}$	$\frac{M\ SNI}{M\ \boldsymbol{Sham}}$	$\frac{F\ Sham}{M\ Sham}$	$\frac{F\ SNI}{F\ Sham}$
*Mrgprx3*	MAS related GPR family member X3	0,43	3,29**	1,63	1,43_NS_
*Atf3*	Activating transcription factor 3	0,16	4,29***	2,94	0,70_NS_
*Il21r*	Interleukin 21 receptor	0,33	3,55***	1,82	1,16_NS_
*Ms4a6a*	Membrane spanning 4-domains A6A	0,48	2,00*	1,36	0,96_NS_
*Pbk*	PDZ binding kinase	0,38	3,04*	1,60	1,15_NS_
*Gpr65*	G protein-coupled receptor 65	0,36	2,65*	1,36	0,95_NS_
*mir146a*	MicroRNA 146a	0,44	1,11_NS_	3,37	0,49**

**Table 6:** Sexually dimorphically expressed genes with a $\frac{F\  SNI/F\  Sham}{M\  SNI/M\  Sham}$ of 1.5 < x < 0.667 in ipsilateral spinal cord (SC) L4-L5 tissue after SNI. $\frac{M\  SNI}{M\  Sham}$, $\frac{F\  Sham}{M\  Sham}$ and $\frac{F\  SNI}{F\  Sham}$ for these genes are also shown. Differential expression (DE) was analysed with DESeq2, False discovery rate (FDR) < 0.05 in either Female (F) SNI vs. F Sham or Male (M) SNI vs. M Sham (n = 7). Not significant (NS) = FDR > 0.05, * = FDR < 0.05

Gene name	Description (Ensembl.org)	$\frac{F\ SNI/F\ Sham}{M\ SNI/M\ Sham}$	$\frac{M\ SNI}{M\ Sham}$	$\frac{F\ Sham}{\mathbf{M}\ Sham}$	$\frac{F\ SNI}{F\ Sham}$
*Lilrb3a*	leukocyte immunoglobulin-like receptor. subfamily B (with TM and ITIM domains). member 3-like	2.87	0.97_NS_	0.57	2.78 *
*Mx1*	myxovirus (influenza virus) resistance 1	2.57	1.03_NS_	0.48	2.64*
*Rps9l1*	ribosomal protein S9	2.47	0.77_NS_	0.65	1.90*
*Fam180a*	family with sequence similarity 180. member A	2.39	0.85_NS_	1.14	2.03*
*Cd40*	CD40 molecule	1.61	1.49_NS_	0.67	2.39*
*Rac2*	Rac family small GTPase 2	1.55	1.35_NS_	0.96	2.08*
*Parvg*	parvin gamma	1.51	1.31_NS_	0.77	1.97*
*Ankrd1*	ankyrin repeat domain 1	0.66	5.85*	1.43	3.83_NS_
*Cd22*	CD22 molecule	0.59	3.13*	1.24	1.86_NS_
*Pbk*	PDZ binding kinase	0.53	3.04*	1.15	1.60_NS_
*Frmpd1*	FERM and PDZ domain containing 1	0.52	1.18_NS_	1.71	0.61*
*Gpr65*	G-protein coupled receptor 65	0.51	2.65*	0.95	1.36_NS_
*Il21r*	interleukin 21 receptor	0.51	3.55*	1.16	1.82_NS_
*Zbtb16*	zinc finger and BTB domain containing 16	0.48	0.98_NS_	2.39	0.47*
*Rbm47*	RNA binding motif protein 47	0.46	2.78*	2.04	1.28_NS_
*Reg3b*	regenerating family member 3 beta	0.46	5.77*	1.31	2.63_NS_
*Ccl2*	C-C motif chemokine ligand 2	0.41	4.68*	1.23	1.94_NS_
*F10*	coagulation factor X	0.41	5.39*	1.95	2.21_NS_

The pathway analyses showed 42 pathways (FDR < 0.05) with altered activity in SC after SNI. Of these, the pathways with the largest significance included the “Chemokine signalling pathway” and the “Cytokine-cytokine receptor interaction.” Five and four pathways in males and females, respectively, showed sexually differentiated activity following SNI. All represented different components of the immune system (Table 7 and supplementary table 2a): 305 and 311 biological processes were altered in females and males respectively (EP < 0.05). Of these, several are highlighted in Table 8 and supplementary table 2a.

**Table 7:** The five pathways with the most significant change per false discovery rate (FDR) (KEGG) altered in ipsilateral spinal cord tissue (L4-L5) 7 days after spared nerve injury (SNI). Comparisons are All SNI vs All Sham (*n* = 14), Male SNI vs male Sham (*n* = 7) and Female SNI vs female Sham (*n* = 7). Analysis conducted with Advaita iPathwayguide, FDR used for multiple comparisons correction

Spinal cord: Pathway name	FDR
All SNI vs. All Sham	
Systemic lupus erythematosus	3.517e-6
Chemokine signalling pathway	6.586e-6
Staphylococcus aureus infection	1.098e-5
Cytokine-cytokine receptor interaction	1.516e-5
Phagosome	1.530e-5
Male SNI vs. Male Sham	
Measles	0.028
Inflammatory bowel disease (IBD)	0.006
Toxoplasmosis	0.009
Platelet activation	0.019
JAK-STAT signalling pathway	0.019
Female SNI vs. Female Sham	
Toll-like receptor signalling pathway	6.769e-4
Viral myocarditis	0.002
Amoebiasis	0.015
Transcriptional misregulation in cancer	0.025

**Table 8:** Selected biological processes (KEGG) with changes in ipsilateral spinal cord (L4-L5) 7 days after SNI. Comparisons are All SNI vs. All Sham (*n* = 14), Male SNI vs. male Sham (*n* = 7) and Female SNI vs. female Sham (*n* = 7). Analysis conducted with Advaita iPathwayguide biological processes tool, Elim pruning (EP) used for multiple comparisons correction, with cut-off value < 0.05. Not significant (NS) = EP > 0.05

Biological processes in the spinal cord		
Biological process	Male EP	Female EP
microglia development	0.036	0.029
microglial cell activation involved in immune response	1.500e-6	4.800e-4
microglial cell activation	0.001	2.000e-4
activated T cell proliferation	0.003	2.400e-5
positive regulation of T cell proliferation	3.300e-5	1.400e-5
regulation of mast cell degranulation	NS	0.003
neuron remodelling	NS	0.009
positive regulation of neuron death	0.009	NS
respiratory burst	NS	4.200e-5

#### Dorsal Root Ganglion Transcriptomics—Experiment II on Day 7

**Fig. 6:**
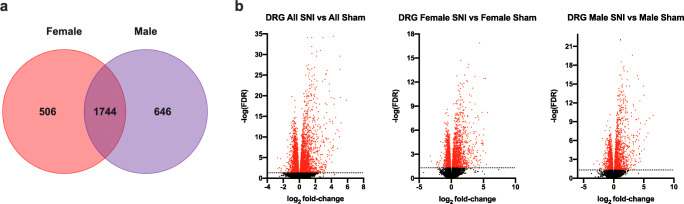
Venn diagram (a) and volcano plots (b) of RNA-Seq data in rat dorsal root ganglion (DRG) following spared nerve injury (SNI) and Sham surgery. All genes included in the Venn diagram have an FDR < 0.05. Volcano plots report log_2_ fold-change and negative logarithmic (base 10) False Discovery Rate (FDR). Dotted line at -log( FDR 0.05), red symbols -log(FDR<0.05). Ipsilateral L4-L5 DRG segment on day 7 after SNI and Sham-surgeries. (*n* = 14: 50% male and female). DESeq2 utilized for DE analysis

A similar analysis of the transcriptome of the DRG was conducted in Experiment II. Compared with the SC transcriptome, more genes showed altered expression in association with SNI in DRG. A comparison of all SNI and sham animals showed 5063 genes with altered expression (FDR < 0.05). The genes with the largest upregulation and downregulation were *lce1* and *tbx22*, respectively (Table 9 and supplementary table 2b). The expression of 2982 and 2842 genes was altered in association with SNI in females and males, respectively, with 1744 genes altered in both sexes (FDR < 0.05) (Fig. 6). In these comparisons, the largest upregulation in males and females occurred in *en1* and *lce1*, respectively, and downregulation in *th* and *tbx22*, respectively.

**Table 9:** Log_2_ Fold-change (FC) for the 25 genes with the largest and smallest differential expression (DE) per log_2_ FC in rat ipsilateral L4-L5 Dorsal root ganglion (DRG) 7 days after spared nerve injury (SNI), and False discovery rate (FDR). SNI and Sham (*n* = 14: 50% male and female). DESeq2 utilized for DE analysis. All reported genes FDR < 0.05

Protein coding genes with the largest upregulation in genes in dorsal root ganglia								
All SNI vs. All Sham DRG			Male SNI vs. Male Sham DRG			Female SNI vs. Female Sham DRG		
Gene name	log_2_ FC	FDR	Gene name	log_2_ FC	FDR	Gene name	log_2_ FC	FDR
*Lce1f*	5.86	1.27E-19	*En1*	6.57	9.88E-11	*Lce1f*	5.65	6.65E-09
*Aabr07042633.1*	5.21	3.96E-14	*Lce1f*	5.93	2.68E-10	*Serpinb2*	5.34	3.55E-13
*Mmp3*	5.12	2.63E-20	*Mmp3*	5.45	6.53E-10	*Loc500712*	5.08	3.55E-13
*Serpinb2*	5.10	2.51E-30	*Aabr07042633.1*	5.41	2.60E-07	*Aabr07042633.1*	5.00	3.92E-06
*Loc500712*	5.07	3.87E-27	*Hamp*	5.18	2.45E-09	*Lipn*	4.98	3.68E-04
*Pdc*	5.00	8.98E-11	*Pdc*	5.08	2.54E-05	*Hsd3b3*	4.95	5.16E-05
*Lipn*	4.75	6.47E-08	*Loc500712*	5.06	6.01E-14	*Pdc*	4.90	7.37E-05
*Hamp*	4.53	1.88E-15	*Hhla1*	5.04	8.90E-07	*Mmp12*	4.84	4.44E-05
*En1*	4.51	1.67E-11	*Serpinb2*	4.91	2.12E-15	*Mmp3*	4.80	5.98E-09
*Ankrd1*	4.32	1.12E-18	*Mmp10*	4.63	4.48E-05	*Xcl1*	4.66	4.69E-05
*Cldn4*	4.32	4.97E-20	*Cldn4*	4.46	1.65E-09	*Reg3b*	4.40	1.33E-17
*Mmp12*	4.24	2.87E-08	*Lipn*	4.42	2.01E-03	*Tacstd2*	4.34	4.03E-06
*Cox6a2*	4.24	3.25E-02	*Ankrd1*	4.36	1.53E-08	*Ankrd1*	4.28	5.22E-08
*Hhla1*	4.23	4.44E-10	*Sprr1a*	4.36	4.83E-13	*Cldn4*	4.18	2.35E-08
*Reg3b*	4.22	3.64E-35	*Cldn7*	4.35	6.76E-04	*Cxcl14*	4.13	1.53E-08
*Cldn7*	4.19	1.94E-07	*Serpina1f*	4.30	1.11E-04	*Smyd1*	4.07	1.92E-02
*Igsf23*	4.16	5.38E-14	*Igsf23*	4.29	7.47E-08	*Cldn7*	3.96	2.07E-03
*Mmp10*	4.15	3.46E-08	*Gzmbl2*	4.29	1.68E-04	*Igsf23*	3.95	6.62E-06
*Tacstd2*	4.13	1.54E-14	*Tgm1*	4.15	3.27E-10	*Mepe*	3.94	3.79E-05
*2310050c09rik*	4.04	2.87E-06	*2310050c09rik*	4.13	3.25E-03	*2310050c09rik*	3.93	6.05E-03
*Rhcg*	4.04	1.23E-04	*Reg3b*	4.09	1.58E-16	*Loc102554842*	3.88	3.07E-05
*Tgm1*	3.98	2.89E-20	*Tacstd2*	3.96	4.24E-08	*Hamp*	3.84	1.26E-05
*Gzmbl2*	3.96	5.18E-08	*Il12rb2*	3.95	4.61E-17	*Il6*	3.83	1.58E-06
*Il6*	3.81	2.00E-14	*Clnk*	3.95	1.99E-07	*Tgm1*	3.78	2.84E-08
*Wt1*	3.78	2.12E-05	*Lsmem1*	3.83	4.92E-10	*Lsmem1*	3.70	5.57E-10
Protein coding genes with the largest downregulation in dorsal root ganglia								
All SNI vs. All Sham DRG			Male SNI vs. Male Sham DRG			Female SNI vs. Female Sham DRG		
Gene name	log_2_ FC	FDR	Gene name	log_2_ FC	FDR	Gene name	log_2_ FC	FDR
*Tbx22*	-2.73	1.20E-05	*Th*	-2.75	1.80E-03	*Tbx22*	-3.29	1.63E-03
*Pinlyp*	-2.56	4.65E-02	*Acot5*	-2.38	6.48E-03	*Gja10*	-2.33	1.69E-02
*Tent5b*	-2.47	2.78E-03	*Mcpt1l1*	-2.22	2.60E-02	*Aabr07033324.1*	-2.28	4.81E-02
*Th*	-2.33	2.02E-05	*Tbx22*	-2.18	2.80E-02	*C1ql2*	-2.20	1.11E-02
*Aabr07006258.1*	-2.30	1.72E-02	*Sstr1*	-1.91	2.96E-03	*Th*	-2.13	8.70E-03
*C1ql4*	-2.25	4.80E-03	*Disp3*	-1.87	4.69E-07	*Ghrl*	-2.05	3.39E-02
*Sim2*	-2.03	4.13E-02	*Loc100909648*	-1.82	8.42E-03	*Tctex1d1*	-1.96	1.69E-02
*Gja10*	-1.68	1.71E-03	*Adra1a*	-1.71	1.95E-04	*Hif3a*	-1.88	1.12E-02
*Acot5*	-1.59	4.74E-03	*Ccdc196*	-1.69	4.54E-03	*Ces2e*	-1.75	6.43E-03
*Ces2e*	-1.58	1.53E-04	*Cav3*	-1.64	8.26E-03	*Col10a1*	-1.66	1.53E-03
*Prr15l*	-1.53	9.97E-07	*Hoxb8*	-1.64	8.54E-04	*Igsf21*	-1.65	2.76E-05
*Igsf21*	-1.52	5.36E-10	*Kcng1*	-1.60	9.81E-04	*Hrh3*	-1.54	5.60E-04
*Gcnt7*	-1.45	4.79E-03	*Dnah6*	-1.58	1.87E-03	*Prr15l*	-1.51	2.88E-03
*Krt27*	-1.44	2.53E-02	*Best3*	-1.56	6.23E-03	*Tmc2*	-1.33	3.83E-02
*Hoxb8*	-1.44	7.25E-06	*Prr15l*	-1.56	2.60E-03	*Klhl14*	-1.30	3.45E-05
*Disp3*	-1.43	2.64E-08	*Siah3*	-1.55	4.74E-03	*Hoxb8*	-1.21	2.28E-02
*Sstr1*	-1.41	1.67E-04	*S100g*	-1.47	1.18E-04	*Mir370*	-1.20	3.71E-02
*Tctex1d1*	-1.37	6.79E-03	*Itga11*	-1.46	1.29E-04	*Best3*	-1.14	2.38E-02
*Rgd1564548*	-1.36	1.10E-02	*Esrrb*	-1.44	5.90E-04	*Zbtb16*	-1.14	7.85E-04
*Rgd1563307*	-1.35	3.84E-02	*Krt28*	-1.42	2.61E-08	*Gjd2*	-1.12	5.46E-03
*Siah3*	-1.33	2.57E-04	*Igsf21*	-1.40	3.01E-04	*Pla2g7*	-1.11	1.02E-04
*Ghrl*	-1.32	2.40E-02	*Gpr3*	-1.39	8.27E-03	*Aabr07064415.1*	-1.11	2.41E-02
*Best3*	-1.31	1.05E-04	*Ankrd66*	-1.39	1.93E-02	*Hapln4*	-1.09	1.31E-07
*Hif3a*	-1.30	1.36E-02	*Aabr07064415.1*	-1.39	4.28E-03	*Tuba8*	-1.08	5.94E-03
*Klrg2*	-1.30	1.15E-02	*Aabr07029269.1*	-1.38	5.04E-06	*Disp3*	-1.07	7.00E-03

Fifty-three genes (FDR < 0.05) from the PainNetworks rat-centric database showed a sexually dimorphic response to SNI in DRG, of these, the genes with the largest Δ $\frac{F\  SNI/F\  Sham}{M\  SNI/M\  Sham}$are shown in Table 10 and supplementary table 2b. However, all genes showed DE associated with SNI in both sexes (FDR < 0.05). Both *Aif1* and *Tyrobp* showed significant increases in expression in both sexes (FDR < 0.05). Of the sexually dimorphically expressed pain genes in DRG, *Abcc3*, *Ccr5*, *P2ry12* and *Tlr4* were also sexually dimorphically expressed in SC tissue. Sexually dimorphically expressed genes with the largest $\frac{F\  SNI/F\  Sham}{M\  SNI/M\  Sham}$ are reported in Table 11 and supplementary table 2b.

**Table 10:** Mean fold change of normalized counts of genes from the PainNetworks rat-centric database in dorsal root ganglion (DRG) with sexually dimorphic expression. Ipsilateral L4-L5 DRG 7 days after spinal nerve injury (SNI) differential expression (DE) analysed with DESeq2. All groups: *n* = 7. False discovery rate < 0.05 = *, < 0.01 = **, < 0.001 = ***, < 0.0001 = **** and > 0.05 = NS

Gene name	Description (Ensembl.org)	$\frac{FSNI/ FSham}{MSNI/ MSham}$	$\frac{MSNI}{MSham}$	$\frac{FSham}{MSham}$	$\frac{FSNI}{FSham}$
*Bhlhe22*	Basic helix-loop-helix family member e22	1,56	1,20***	0,57	1,87_NS_
*C5ar1*	Complement C5a receptor 1	2,38	0,76_NS_	0,62	1,80*
*Ccr5*	C-C motif chemokine receptor 5	2,55	0,99_NS_	0,40	2,52***
*Cd274*	CD274 molecule	1,13	0,99****	0,79	1,12_NS_
*Chrna4*	Cholinergic receptor nicotinic alpha 4 subunit	0,83	0,87*	1,21	0,72_NS_
*Cnr2*	Cannabinoid receptor 2	1,88	0,96_NS_	0,58	1,81*
*Dpp4*	Dipeptidyl peptidase 4	2,75	0,62_NS_	0,58	1,71*
*Grin2a*	Glutamate ionotropic receptor NMDA type subunit 2A	1,26	1,32*	0,90	1,67_NS_
*Hrh3*	Histamine receptor H3	0,49	0,95_NS_	1,15	0,47***
*Prokr1*	Prokineticin receptor 1	1,03	1,04***	0,99	1,07_NS_
*Thbs4*	Thrombospondin 4	2,23	0,70_NS_	0,61	1,57**

**Table 11:** Sexually dimorphically expressed genes with fold change (FC) $\frac{F\  SNI/F\  Sham}{M\  SNI/M\  Sham}$ 2.5 < × < 0.4 in ipsilateral L4-L5 Dorsal root ganglion after SNI. $\frac{M\  SNI}{M\  Sham}$, $\frac{F\  Sham}{M\  Sham}$ and $\frac{F\  SNI}{F\  Sham}$ of these genes also shown. Differential expression (DE) analysed with DESeq2, False discovery rate (FDR) < 0.05 in either Female (F) SNI vs. F Sham or Male (M) SNI (n = 7). * = FDR < 0.05

Gene name	Description (Ensembl.org)	$\frac{FSNI/ FSham}{MSNI/ MSham}$	$\frac{MSNI}{MSham}$	$\frac{FSham}{MSham}$	$\frac{FSNI}{FSham}$
*Mcpt1l1*	mast cell protease 1-like 1	9.66	0.21*	0.22	2.06_NS_
*Cxcr2*	C-X-C motif chemokine receptor 2	5.34	0.76_NS_	0.53	4.05*
*Asb2*	ankyrin repeat and SOCS box-containing 2	4.61	0.79_NS_	0.48	3.65*
*Il1b*	interleukin 1 beta	4.48	1.41_NS_	0.32	6.31*
*Ctla4*	cytotoxic T-lymphocyte-associated protein 4	4.44	1.38_NS_	0.48	6.14*
*Dntt*	DNA nucleotidylexotransferase	4.27	1.64_NS_	0.34	7.01*
*Exoc3l4*	exocyst complex component 3-like 4	4.26	0.64_NS_	0.47	2.72*
*Prf1*	perforin 1	4.20	1.30_NS_	0.43	5.47*
*Bmp4*	bone morphogenetic protein 4	4.18	0.55_NS_	0.50	2.30*
*Pla2g2d*	phospholipase A2. group IID	3.87	1.05_NS_	0.27	4.08*
*Plac8*	placenta associated 8	3.73	0.76_NS_	0.51	2.84*
*Gpr84*	G protein-coupled receptor 84	3.63	1.23_NS_	0.35	4.47*
*Tlr5*	toll-like receptor 5	3.53	0.70_NS_	0.57	2.48*
*Tekt3*	tektin 3	3.16	0.85_NS_	0.57	2.69*
*Acot5*	acyl-CoA thioesterase 5	3.10	0.19*	0.51	0.58
*Slitrk6*	SLIT and NTRK-like family. member 6	3.01	1.32_NS_	0.55	3.96*
*Prg4*	proteoglycan 4	3.00	1.11_NS_	0.53	3.34*
*Tmem45al*	transmembrane protein 45A-like	2.98	1.33_NS_	0.37	3.97*
*Lst1*	leukocyte specific transcript 1	2.75	1.25_NS_	0.29	3.44*
*F3*	coagulation factor III. tissue factor	2.63	0.80_NS_	0.59	2.12*
*Prox1*	prospero homeobox 1	2.58	0.94_NS_	0.56	2.42*
*Sox9*	SRY box 9	2.54	0.84_NS_	0.43	2.14*
*Cd244*	CD244 molecule	2.53	1.80_NS_	0.39	4.54*
*Lilrb3a*	leukocyte immunoglobulin-like receptor. subfamily B (with TM and ITIM domains). member 3-like	2.53	1.44_NS_	0.51	3.63*
*Foxd1*	forkhead box D1	2.52	0.82_NS_	0.79	2.06*
*Ghrl*	ghrelin and obestatin prepropeptide	0.38	0.63_NS_	1.34	0.24*
*Hif3a*	hypoxia inducible factor 3 subunit alpha	0.37	0.75_NS_	2.49	0.27*
*Cyp27b1*	cytochrome P450. family 27. subfamily b. polypeptide 1	0.35	10.14*	1.44	3.52_NS_
*Ptx3*	pentraxin 3	0.34	4.28*	1.12	1.46_NS_
*Eme1*	essential meiotic structure-specific endonuclease 1	0.29	4.64*	1.72	1.32_NS_
*Neu2*	neuraminidase 2	0.29	4.34*	2.48	1.24_NS_
*Mir370*	microRNA 370	0.24	1.85_NS_	2.08	0.44_*_
*Otol1*	otolin 1	0.23	5.45*	2.68	1.26_NS_
*C1ql2*	Complement C1q like 2	0.20	1.13_NS_	2.28	0.22*
*Trpv6*	Transient receptor potential cation channel. subfamily V. member 6	0.17	4.09*	3.51	0.69_NS_

Forty-nine pathways with altered activity associated with SNI were identified. The most significant were “Haematopoietic cell lineage” and “Cell adhesion molecules” (FDR < 0.05). No pathways were altered in males only, while 11 were altered in females only. Among these were “Cell adhesion molecules” and “Phagosome” (Table 12). Both sexes displayed many altered biological processes, several of interest being shown in (Table 13).

**Table 12:** The five pathways with the most significant change, measured with False discovery rate (FDR) (KEGG), in ipsilateral dorsal root ganglion (DRG) L4-L5 7 days after SNI. Comparisons are All SNI vs. All Sham (*n* = 14), Male SNI vs. Male Sham (*n* = 7) and Female SNI vs. Female Sham (*n* = 7). No Male-only changes were reported. Analysis was conducted with Advaita iPathwayguide, FDR was used for multiple comparisons correction

DRG: Pathway name	FDR
All SNI vs. All Sham	
Haematopoietic cell lineage	1.785e-11
Cell adhesion molecules	6.130e-10
Systemic lupus erythematosus	1.072e-6
Phagosome	5.770e-6
Cytokine-cytokine receptor interaction	5.770e-6
DRG Female SNI vs. Female Sham	
Malaria	4.749e-4
Cell adhesion molecules	9.765e-4
Tuberculosis	0.003
Viral Myocarditis	0.003
Phagosome	0.016

**Table 13:** Selected biological processes (KEGG) with changes in ipsilateral dorsal root ganglion (L4-L5) 7 days after SNI. Comparisons are All SNI vs. All Sham (*n* = 14), Male SNI vs. male Sham (*n* = 7) and Female SNI vs. female Sham (*n* = 7). Analysis conducted with Advaita iPathwayguides biological processes tool, Elim pruning (EP) used for multiple comparisons correction, with cut-off value < 0.05. Not significant = EP > 0.05

Biological processes in dorsal root ganglia		
Biological process	Male EP	Female EP
regulation of sensory perception of pain	0.023	0.022
positive regulation of T-helper cell differentiation	NS	0.0014
T cell activation involved in immune response	0.027	0.021
T cell mediated immunity	NS	0.037
T cell migration	NS	0.039
T cell apoptotic process	0.031	NS
T cell chemotaxis	0.030	NS
regulation of alpha-beta T cell differentiation	NS	0.021
regulation of T helper 1-type immune response	NS	0.003
positive regulation of T helper 1 cell cytokine production	0.017	0.008
T helper 1 cell differentiation	NS	0.008
positive regulation of T helper 2 cell cytokine production	0.047	0.020
regulation of T helper 2 cell differentiation	NS	0.031
positive regulation of T helper 2 cell differentiation	0.017	NS
T helper 2 cell differentiation	0.047	NS
T helper 17 cell differentiation	NS	0.031
positive regulation of T helper 17-type immune response	0.047	0.020
B cell receptor signalling pathway	NS	1.400e-4
negative regulation of B cell proliferation	NS	0.001
positive regulation of B cell proliferation	0.004	4.100e-4
negative regulation of B cell receptor signalling pathway	NS	0.008
regulation of B cell receptor signalling pathway	0.011	0.007
regulation of microglial cell migration	NS	0.020
microglial cell activation	NS	0.002
neuronal action potential	NS	0.022
regulation of neuronal synaptic plasticity	0.036	NS
positive regulation of synaptic transmission, glutamatergic	0.006	0.040
regulation of synaptic transmission, GABAergic	0.016	NS

## Discussion

In this study, we set out to characterize sex differences in behaviour, immunohistochemical markers, protein content, and RNA expression in both male and female rats, following spared nerve injury. Compared with males, female rats demonstrated consistently greater and more rapidly developing mechanical allodynia. The decrease in the number of neurons positive for CGRP and IB-4 in the DRG was equal in both sexes, suggesting injury of similar magnitude in both sexes. The increase in staining of glial cell markers IBA1 and GFAP in the SC was fairly consistent and did not reveal convincing sexual dimorphism on Day 21 after injury. No changes to the proteome of the CSF due to SNI could be demonstrated at 21 days. Transcriptomics analyses revealed DE in several pain-related genes and others of interest in the two sexes at Day 7 after SNI in DRG and/or SC, among the most interesting being *Ctla4*, *Cd274*, *Dpp4*, *Il1b*, *Cxcr2*, *Thbs4*, *Hrh3*, *Pdyn*, and *Bhlhe22*.

### Behavioural Tests

In both experiments, behavioural tests revealed sexual dimorphism between male and female behaviour post-surgery. Female rats were consistently more sensitive to von Frey filaments, with the results implying more rapid and stronger development of mechanical allodynia. In the acetone test, a similar consistent sexually dimorphic response was seen in Experiment I on Day 7. However, in Experiment II, the number of positive responses was approaching the maximum possible in both sexes at that time point and no conclusions about sex differences could be made. Previous studies have reported varying results in sex differences in behaviour following PNI. For example, greater mechanical allodynia in female rats has been reported in at least one PNI study and, in another study, following CCI of the infraorbital nerve [8, 40]. In two other studies, no behavioural differences were reported, even though differences in the mechanisms of putative sex differences were hypothesized [9, 10]. In contrast to our study, one study found that male rats demonstrated lower mechanical force thresholds for up to 17 days, but not later, after CCI [11]. These results highlight the complex nature of behavioural findings as a factor of time. Our behavioural findings, greater static mechanical allodynia in females following SNI in two separate experiments, lay an interesting basis for analyses of possible differences in IHC, proteomics and transcriptomics, which could clarify some of the mechanisms underlying observed behavioural sex differences.

### Immunohistochemistry

IHC was conducted to assess whether neuronal injury, as measured by the loss of IB-4 and CGRP-positive neurons in DRG, was similar in both sexes, and whether GFAP and IBA1 IHC of the SC would show sex differences that, in turn, might explain the observed sex differences in behaviour.

IB-4- and CGRP-immunostaining of DRG following SNI showed a robustly lower percentage of positively-staining CGRP- and IB4- cells in both sexes, with no significant sex differences. CGRP-positive DRG neurons are traditionally considered as peptidergic neurons, whereas IB4-positive cells are non-peptidergic [41]. IB-4- and CGRP-staining has been shown to decrease following PNI [42]. The fact that the changes were similar in both sexes indicates that nerve injury was of comparable magnitude in both sexes, which implies that sex differences in NP probably arise at later stages of nociception or pain perception than at the level of the injury. This is line with prior research on RNA-seq of DRG neurons, which showed only few sexually dimorphically expressed genes after PNI [43].

SNI potently increased the expression of IBA1 in animals of both sexes. No sex-specific differences in SNI-induced IBA1 expression in either L4 or L5 regions of SC were observed. SNI increased the expression of GFAP in both male and female rats, with no consistent sexual dimorphism. IBA1 and GFAP expression was analysed with IHC in samples collected on Day 21, while the major sex-related differences in pain-related behaviour were seen already by Day 7 after SNI. However, on Day 7, *Aif1*, the gene coding for IBA1, did not show sexually dimorphic DE in SC transcriptomics, supporting the notion of there being no sex difference in microgliosis. In line with our results, previous studies in mice show a similar increase in IBA1-staining in both sexes following PNI [10]. However, the mechanisms that lead to the development of NP in the two sexes seem to differ at another level of control. While P2X4R-signalling in microglia is necessary for the development of NP in males, females might, in addition, develop NP through pathways relying on cells of the adaptive T cell-mediated immune system [9, 14] [15] [12, 13]. Thus we conclude that, even though microglia may be important for the development of NP, anti-IBA1-immunoreactivity does not associate with sexually dimorphic behavioural outcomes.

### CSF Proteomics

To the best of our knowledge, the CSF proteome of male and female rats following SNI, or PNI in general, has not previously been characterized. Here, we show that the amounts of individual proteins were not significantly altered by SNI, even though sex differences were observed at baseline and dimorphism in behaviour and gene expression was already observed by Day 7. The CSF samples analysed were not collected until 21 days after injury, allowing for the possibility that some acute and short-lasting changes were not captured.

The role of CSF proteins in NP remains of interest because CSF, unlike DRG or SC tissue, is available from patients. Human studies suggest changes in CSF proteomics in NP patients [44]. For example, chemokines have been shown to be altered in patients who have had NP for at least 6 months. However, it is possible that changes in human CSF proteomics are associated with comorbidities that predispose to NP [45, 46].

### Transcriptomics

In Experiment II, we set out to find transcripts, whose change in expression might explain the observed behavioural sex differences in NP on Day 7 after SNI, by analysing gene expression in both DRG and SC.

FCs in DRG were generally greater than those in the SC, reflecting the role of DRG neurons as a primary site of the production of proteins in response to PNI. In general, female DRG and SC showed more genes with DE after SNI compared with sham, than males. Altogether 2333 genes were sexually dimorphic in DRG in our study. A previous study, reported 1513 sexually dimorphic genes in response to CCI, 14 days after injury, with more changes in males than in females [19], in contrast to our results. Our earlier time point of 7 days and behavioural data consistently showing greater mechanical allodynia in females imply a divergent change in transcription in DRG early in the course of development of NP. In another study, in which SC RNA-seq was carried out on day 7 after SNI on male rats, however, with smaller group sizes, the top 20 up- and downregulated genes were reported. Of these 12, *Atf3, C1qa, C2, Ccl2, Clec4a1, Csf1r, Ctss, Cyth4, Fcrl2, Ly86, Reg3b, Tlr7,* were upregulated in our dataset, representing a significant concordance of the datasets. Interestingly, none of the downregulated genes were downregulated in our results [18]. In the same study, RNA-Seq was analysed also on Day 3 and 14, with clearly different profiles in differentially expressed genes, further implying the importance of the timing of sample extraction and the dynamic nature of transcriptional changes in NP.

In a meta-analysis of microarray studies for pain in rodents, the two most reliably upregulated genes in DRG after PNI were *Reg3b* and *Ccl2* [47]. These two genes were also upregulated in both male and female DRG in our data, with no sex differences.

To the best of our knowledge, no previous study has assessed sexually dimorphic gene expression changes in SC after PNI. As further support for the role of *Reg3b* and *Ccl2,* the genes were also upregulated in SC in our study but, interestingly, only in males (FDR < 0.05). In addition, the gene displaying the largest upregulation in SC, in both sexes, was *Cxcl13,* also shown to be upregulated following SNL and to be relevant for the activation of astrocytes and for driving NP [48]. As discussed earlier, microgliosis occurred in both sexes on account of IBA1, the product of *Aif1*. However, the pathway analyses show alteration in several microglia-relevant pathways in females only in the DRG.

Pathway and biological process analysis revealed, in particular, several T cell and other immunology-related findings. Neuronal plasticity and related processes, such as apoptosis, may also be crucial in the development of pathological activity and sensitization in the sensory system. As we were particularly interested in genes that might explain the behavioural findings, we also focused on genes retrieved from the PainNetworks database. Many of these genes demonstrated significant sexually dimorphic responses in DRG, and/or lesser dimorphic responses in SC, after SNI. We also discuss other genes with a large $\frac{F\  SNI/F\  Sham}{M\  SNI/M\  Sham}$that have been linked to pain, as well as other genes that are completely novel in this respect. We categorized our findings into several groups, aware that genes may fall into more than one category: genes related to T cells (*Cd28*, *Ctla4*, *Cd274*, *Cd4*, *Prf1*); other immunological responses, (*Dpp4*, *C5ar1*, *Cxcr2* and *Il1b*); neuronal transmission (*Hrh3*, *Thbs4*, *Chrna4* and *Pdyn*); plasticity (*Atf3, C1qc* and *Reg3b*); and other genes with significant sexually dimorphic changes (*Bhlhe22*, *Mcpt1l, Trpv6)*.

#### Changes Related to T Cells

Several immune-related pathways and biological processes were altered in DRG and/or SC in both sexes. We identified numerous T-cell-related transcripts that showed altered expression. T cells are paramount regulators and effectors in immune responses, and they have been postulated to play a part in sex differences in pain, and to be of greater importance in females [9, 10].

One interesting T cell–related gene was *Cd274,* which displayed male-only upregulation in DRG. The gene *Cd274* is translated into the protein PD-L1, which downregulates effector CD8+ T cell activity [49]. Interestingly, PD-L1 is also produced in DRG neurons and it has been reported to have potent antinociceptive effects in mice through its receptor PD-1 [50]. As PD-L1 may exert an antinociceptive effect, lack of its upregulation in females might contribute to sex differences in mechanical allodynia.

Other genes indicating a stronger female T cell response in the DRG were *Cd4*, *Prf1*, and *Dntt*. The gene *Cd4*, the product of which is the prototypic marker for helper T cells, showed an increase in expression in females only (log_2_ FC 1.29, FDR <0.05). Upregulation of *Prf1* also occurred in females only (log_2_ FC 2.47, FDR, 0.05). The gene *Prf1* codes for perforin, which forms cytolytic pores and is a key end-stage effector mechanism of CD8+- and NK-cells [51]. Further, we demonstrate a female-only upregulation of *Dntt*, a lymphocyte-specific-DNA polymerase involved in adding nucleotides to T-cell receptors, which are essential for the function of T cells [52, 53].

Two additional T cell–related transcripts of great interest that were upregulated in the DRG are *Ctla4* (log_2_ FC = 2.6 upregulation in females) and *Cd28* (log_2_ FC = 2.1 upregulation in males). Binding of *ctla4* on T cells by CD80/86 on antigen-presenting cells (APC) arrests the cell cycle of T cells, while binding of *cd28* on T cells with the same CD80/86 on APC leads to increased proliferation and increases in effector mechanisms of T cells [54–57]. This finding initially seems to contradict our findings regarding *Cd4*, *Prf1*, and *Dntt,* which indicated a stronger T cell response in females. However, *ctla4* has been shown to be upregulated following CD8+ activation and, as such, the *Ctla4* increase may be secondary to the prior activation of T cells [58]. This hypothesis relies on the other T cell findings described above. Interestingly, CTLA4-inhibitors are in clinical use as immunotherapeutics for their function of activating host immune response against tumours [59]. Thus, the role of CTLA-4 inhibitors in the development of NP in cancer patients would be of interest.

To summarize, T cell–related transcripts show large differences between the sexes, with several changes pointing to T cells having a greater importance in female than in male rats in NP, in line with the findings of Sorge et al [10].

#### Other Changes to the Immunological Response

Other genes closely related to immunological processes were profoundly affected. Of these, *Dpp4*, *C5ar1*, *Cxcr2*, *Il1b* will be discussed.

The gene *Dpp4* is a PainNetworks gene that displays a female-only upregulation following SNI and has been shown to be expressed in spinal astrocytes and microglia, as well as in T cells [60, 61]. Spinal application of DPP4-inhibitors, which are widely used clinically in the treatment of diabetes mellitus [62], decreases mechanical allodynia in inflammatory pain and shows a modest antihyperalgesic effect following partial SNL in male rats [61, 63]. Upregulation of *Dpp4* might thus partly explain the observed sex difference in mechanical allodynia. This opens an opportunity to test the hypothesis that DPP-4-inhibitors might produce an antinociceptive effect in females.

Another prominent, female-specific, upregulated gene is *Cxcr2*, which encodes the receptor binding several chemokines, one of which being CXCL1 [64]. Both *Cxcr2* and *Cxcl1* have been shown to be upregulated in pain states in DRG [65, 66]. The expression of *Cxcl1* was not altered in our experiment but CXCR2-signalling has previously been linked to maintenance of inflammatory pain [67]. Interestingly, CXCR2 antagonists have demonstrated an antinociceptive effect in the CCI model of NP [68]. Therefore, the female-specific upregulation of *cxcr2* suggests that CXCR2 antagonists might be more efficacious for NP in females.

The complement cascade genes *C1qc* and *C3* were upregulated in the DRG of both sexes. Downstream in the complement cascade of the protein products of these genes is the anaphylotoxin C5a, which is the ligand for the receptor C5ar1. This receptor is coded by the gene *C5ar1*, which was upregulated in DRG only in females. C5a and its receptors have previously been shown to be increased in CCI-, SNL- and SNI-operated rats and inhibition of C5a has been shown to aggravate development of NP in male rats [69]. What function this larger upregulation serves in females remains undetermined and it would be interesting to compare C5a responses in males and females.

*Il1b*, encoding the protein Interleukin-1β, was prominently upregulated in female DRG (FC = 6.31, FDR < 0.05) compared with males (FC = 1.41, FDR > 0.05). *Il1b* has been shown to be of importance in the development of NP [70, 71]. Notably, prior studies have mostly been performed in male rodents and we did not find a significant change in the upregulation of *Il1b* in male rats. This may be due, at least partly, to the strict statistical correction for multiple comparisons. The large upregulation in females might explain some of the observed difference in the pain behaviour between females and males. Several drugs target *Il1b* signalling. One of them is anakinra, an IL1 receptor antagonist. This offers a possibility for further studies of sex-specific indications for drugs in this group [72].

#### Neuronal Transmission

Several ion channels and neurotransmitter systems are important in the pathophysiology of NP and targets for its treatment. Ca_v_α_2_δ_1_, for which Thrombospondin 4 is the endogenous ligand, α7 nicotinic receptor and histamine 3 receptors have been studied as target proteins for drugs for NP [73–75]. Interestingly, the biological process “neuronal action potential” was only altered in females while “regulation of synaptic transmission, GABAergic” showed a change only in males, and “positive regulation of synaptic transmission, glutamatergic” was altered in both sexes.

Interestingly, we observed an increase of *Thbs4* in female, but not in male DRG. Thrombospondin 4 is an endogenous ligand of Ca_v_α_2_δ_1_ and is implicated in central sensitization [74]. Moreover, Ca_v_α_2_δ_1_ is also the target protein for pregabalin and gabapentin, both of which are first-line drugs for NP [76]. As a result of pregabalin or gabapentin binding to this receptor subunit, the calcium channel does not locate to the cell membrane, leading to inhibition of the release of presynaptic glutamate [77, 78]. As Thbs4 antibodies have been shown to exert antihyperalgesic effects, upregulation of *Thbs4* in females might explain some of the observed increased mechanical allodynia in females. Prior studies have reported varying changes in expression of *Thbs4*, with both upregulation and downregulation being described in males, depending on the method used. Importantly, females have not been studied before [79–81]. We are not aware of any studies on gender differences in treating NP with pregabalin or gabapentin in humans.

The gene *Chrna4*, coding for the neuronal acetylcholine receptor subunit alpha-4 (nAChRα4), displayed significant downregulation in DRG in males only. Previously, CCI of the infraorbital nerve in rats showed greater mechanical allodynia and increased expression of *Chrna4* in females [40]. With an overall sex-dependent change in the same direction, and the fact that nicotinic receptors (of which nAChRα4 is one) mediate fast depolarisations at synapses [82], upregulation of *Chrna4* might sensitize neurotransmission in a sex-specific manner.

The gene *Hrh3*, coding for the presynaptic inhibitory histamine 3 autoreceptor (H_3_ receptor) (listed in the PainNetworks database) shows dramatic downregulation exclusively in females. The evidence on the role of H_3_ receptors in NP is, however, conflicting. Most studies seem to suggest that H_3_ receptor antagonists and inverse agonists mediate antinociception [75, 83], so this finding might not directly explain the observed sex difference in mechanical allodynia. However, some studies have shown H_3_ agonists to have antinociceptive effects [84] and therefore, as one of the few PainNetworks genes displaying downregulation only in females, *Hrh3* is of future interest.

The gene *Pdyn*, coding for prodynorphin, showed a male versus female sex FC ratio of 1.44, the most substantial change in expression amongst the PainNetworks genes in the SC. Prodynorphin is the precursor to dynorphin [85] and dynorphin-containing interneurons have been shown to inhibit excitatory somatostatin-containing interneurons in SC [86]. These somatostatin-containing neurons have been suggested to mediate Aβ-fibre-mediated sensitization of projection neurons [86]. In our study, both sexes displayed an increased expression of *Pdyn* in SC, although the increase was larger in males. Greater upregulation of prodynorphin in males would suggest stronger inhibition of projection neurons which could partly explain the observed behavioural finding.

#### Plasticity

Both functional and structural plasticity may be important for the development of NP [86, 87]. Although the exact mechanism of maladaptive structural remodelling and reorganization is not known, various genes related to apoptosis and neuronal remodelling may play an essential role in the formation of aberrant connections in the SC. C1q is primarily linked to complement cascade but may affect synaptic pruning [88]. Expression of C1q subunits was upregulated at the spinal level in both males and females, supporting the idea of a role for C1q in SNI-induced plasticity/remodelling. The previously discussed thrombospondin 4 also promotes excitatory synaptogenesis and may therefore affect plasticity, especially in females. This in turn might explain the larger mechanical allodynia in these [74, 89]. In addition, the expression of many genes related to the biological process “Neuron apoptotic process” were found to be differentially expressed in SC tissue: *Reg3b*, *Ccl2* and *Grn* (progranulin) were upregulated only in males, *Coro1a* only in females, and *Cx3cr1* (the gene for CX3CR1, also known as fractalkine receptor), *Ctsz* and *Tyrobp* in both males and females. The gene *Atf3,* coding for the transcription factor ATF3, is regarded as a marker of neuronal injury, but also of neuronal regeneration [90, 91]. Previous studies have also found upregulation of *Atf3* in both DRG and SC in males [92]. The effect of male-only upregulation of *Atf3* and *Reg3b* in SC on behavioural sex difference remains to be studied.

#### Other

Genes displaying a sexually dimorphic response in the DRG were more plentiful and changes were larger than in the SC. The gene *Mcpt1i1,* displayed the largest change in females compared with males and the gene *Trpv6* showed the largest male vs. female change in ratio in the DRG.

The gene *Trpv6*, encoding TRPV6 (a highly-selective Ca^2+^ channel), is expressed in epithelial cells [93, 94] but expression in neural tissue has also been reported [95]. TRPV6 has been studied as a tumour marker and target [96] but its role in nociception and NP remains unknown.

The gene *Mcpt1i1,* coding for mast cell protease 1-like 1 displayed the largest female versus male upregulation in DRG. This enzyme and its functions are poorly understood and they have not been linked to NP. However, it is worth considering whether it could have a similar function to mast cell protease 1, which converts angiotensin 1 into angiotensin 2 [97]. Angiotensin 2 and the angiotensin 2 receptor in DRG have been linked to NP. If the protein product of *Mcpt1l1* had a similar function, it could explain some of the observed sex difference and would suggest a sex-specific efficacy for AT2-receptor blockers, which have been studied as drugs for NP [98, 99].

The gene *Bhlhe22* displayed the largest change of all PainNetworks genes. It also showed a sexually dimorphic response in the DRG, with a 5-fold increase in males. The absence of its transcription factor (Bhlb5) causes loss of DH interneurons which normally inhibit itch modulated by dynorphin [100, 101]. However, the possible role of *Bhlhe22* in the modulation of nociception/sensory systems at the DRG level remains to be studied.

## Conclusions

Many of the most disparate sex differences in expression following SNI involve components of the immune system, and lymphocytes in particular, in combination with a repeatedly more significant mechanical allodynia in females. The microglial or astrocytic proliferation and activation do not reveal a dichotomous role for these cells in either sex under these circumstances in the SC, nor did the proteome of CSF reveal new insights. The results strengthen the notion that mechanisms behind NP may significantly differ between the sexes and underline the importance of different immunologic mechanisms of NP. Our research of DRG and SC transcriptomics provides insight into ideas for new interventional studies to test the hypotheses that were laid out and to characterize further the increasingly precise sexually dimorphic mechanisms of neuropathic pain. We highlight the sexually dimorphic expression of the genes *Thsb4, Ctla4 And Dpp4*, as these are directly affected by drugs which are clinically available and thus lend themselves as prime targets for interventions in future studies. Our results also indicate that greater emphasis should be put on gender differences in clinical research and drug development for the treatment of NP.

## Supplementary Information

ESM 1 (EPS 160 kb)

ESM 2 (XLSX 11 kb)

ESM 3 (XLSX 53 kb)

## Data Availability

The datasets used and/or analysed during the current study are available from the corresponding author on reasonable request.
